# Magnetic Mode Coupling in Hyperbolic Bowtie Meta-Antennas

**DOI:** 10.1021/acs.jpclett.3c01620

**Published:** 2023-08-25

**Authors:** Sema Ebrahimi, Alina Muravitskaya, Ali M. Adawi, Anne-Laure Baudrion, Pierre-Michel Adam, Jean-Sebastien G. Bouillard

**Affiliations:** † Light, Nanomaterials, and Nanotechnologies Laboratory, CNRS EMR 7004, University of Technology of Troyes, F-10004 Troyes Cedex, France; ‡ Department of Physics and Mathematics, University of Hull, Cottingham Road, Hull HU6 7RX, United Kingdom; § G.W. Gray Centre for Advanced Materials, University of Hull, Cottingham Road, Hull HU6 7RX, United Kingdom

## Abstract

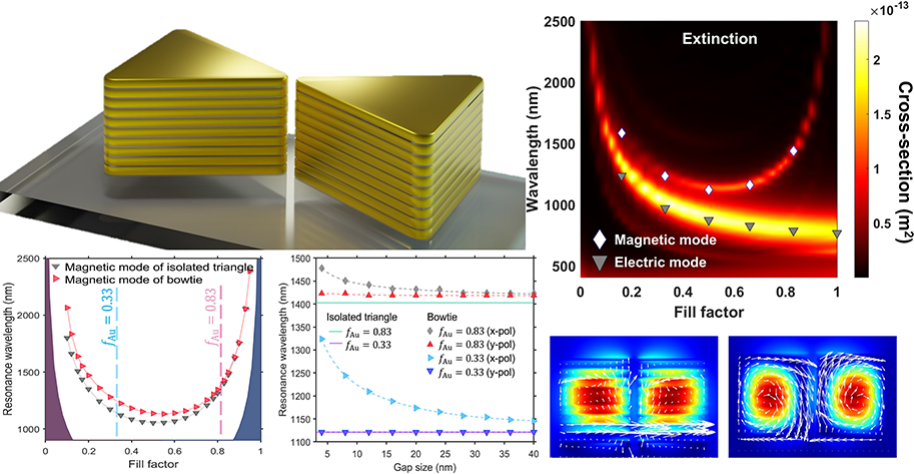

Hyperbolic metaparticles have emerged as the next step in metamaterial applications, providing tunable electromagnetic properties on demand. However, coupling of optical modes in hyperbolic meta-antennas has not been explored. Here, we present in detail the magnetic and electric dipolar modes supported by a hyperbolic bowtie meta-antenna and clearly demonstrate the existence of two magnetic coupling regimes in such hyperbolic systems. The coupling nature is shown to depend on the interplay of the magnetic dipole moments, controlled by the meta-antenna effective permittivity and nanogap size. In parallel, the meta-antenna effective permittivity offers fine control over the electrical field spatial distribution. Our work highlights new coupling mechanisms between hyperbolic systems that have not been reported before, with a detailed study of the magnetic coupling nature, as a function of the structural parameters of the hyperbolic meta-antenna, which opens the route toward a range of applications from magnetic nanolight sources to chiral quantum optics and quantum interfaces.

Metamaterials constitute an emerging class of materials with exotic optical properties enabling a wide range of technological applications such as negative refraction,^1,2^ optical cloaking,^3,4^ super-resolution imaging,^5^ ultracompact optical circuit elements,^6^ and efficient energy harvesting.^7−14^ Designing and engineering metamaterials opens new avenues to manipulate electromagnetic waves, overcoming the constraints of natural materials. Hyperbolic metamaterials, an emerging class of highly anisotropic metamaterials, have recently attracted a lot of attention due to the highly controllable electromagnetic properties they provide. Their hyperbolic dispersion not only allows these materials to support high-k modes but also represents a significant increase in the photonic density of states to engineer light–matter interactions.^15−17^ In parallel, their intrinsically anisotropic permittivity offers new ways to achieve very high refractive index structures beyond what is available in nature.^7,15,18,19^ Consequently, hyperbolic metamaterials underpin a vast range of applications such as single-antenna biosensing, plasmonic-based lasing, photovoltaics, and hot-electron generation technologies.^7,20−24^ The two main strategies to realize hyperbolic metamaterials rely on either alternating metal and dielectric layers, or metallic nanowires in a dielectric host.^7,8,21,25−30^ Nanostructuring of bulk hyperbolic metamaterials to create metaparticles offers new possibilities, promising many novel exotic applications.^16,24,31−34^

Recent studies have shown that isolated hyperbolic metaparticles built from alternating metal-dielectric multilayers support well-separated electric and magnetic resonances,^16,33−36^ with the origin of magnetic resonances either attributed to the complex coupling between electric and magnetic multipoles through a multipole decomposition,^33^ or explained in terms of Mie resonances arising from the high values of the perpendicular permittivity component (normal direction to the layers) achievable in hyperbolic metamaterials.^36^ Maccaferri et al.^16^ explored the interplay between the electric and magnetic mode in isolated cylindrical metaparticles allowing fine control over the scattering and absorption properties of the nanostructure. On the other hand, Czajkowski et al.^33^ present a detailed theoretical analysis of the electric and magnetic mode structure of isolated hyperbolic spherical nanoantennas.

Up to now, however, the study of hyperbolic nanostructures has been restricted to isolated metaparticles. Leveraging the coupling of metamaterial nanostructures will unlock novel optical phenomena unavailable in isolated metaparticles, making it possible to capitalize on the full range of opportunities such systems offer.

In this work, we introduce a hyperbolic meta-antenna based on the coupling of two triangular multilayer gold-TiO_2_ nanostructures in a bowtie geometry (Figure 1a) and numerically study in detail the mode structure and rich coupling regimes in such systems, capitalizing on the strong electric field confinement and enhancement traditionally offered by such geometries. Bowtie nanoantennas have been extensively studied in the purely plasmonic regime, including more complex geometries such as core–shell bowtie geometries^37^ and metal–insulator–metal–insulator–metal^32^ geometries. Whereas plasmonic nanoantennas solely support coupling between electric modes, the hyperbolic meta-antenna presented here is shown to support both electric and magnetic dipolar modes.

**Figure 1:**
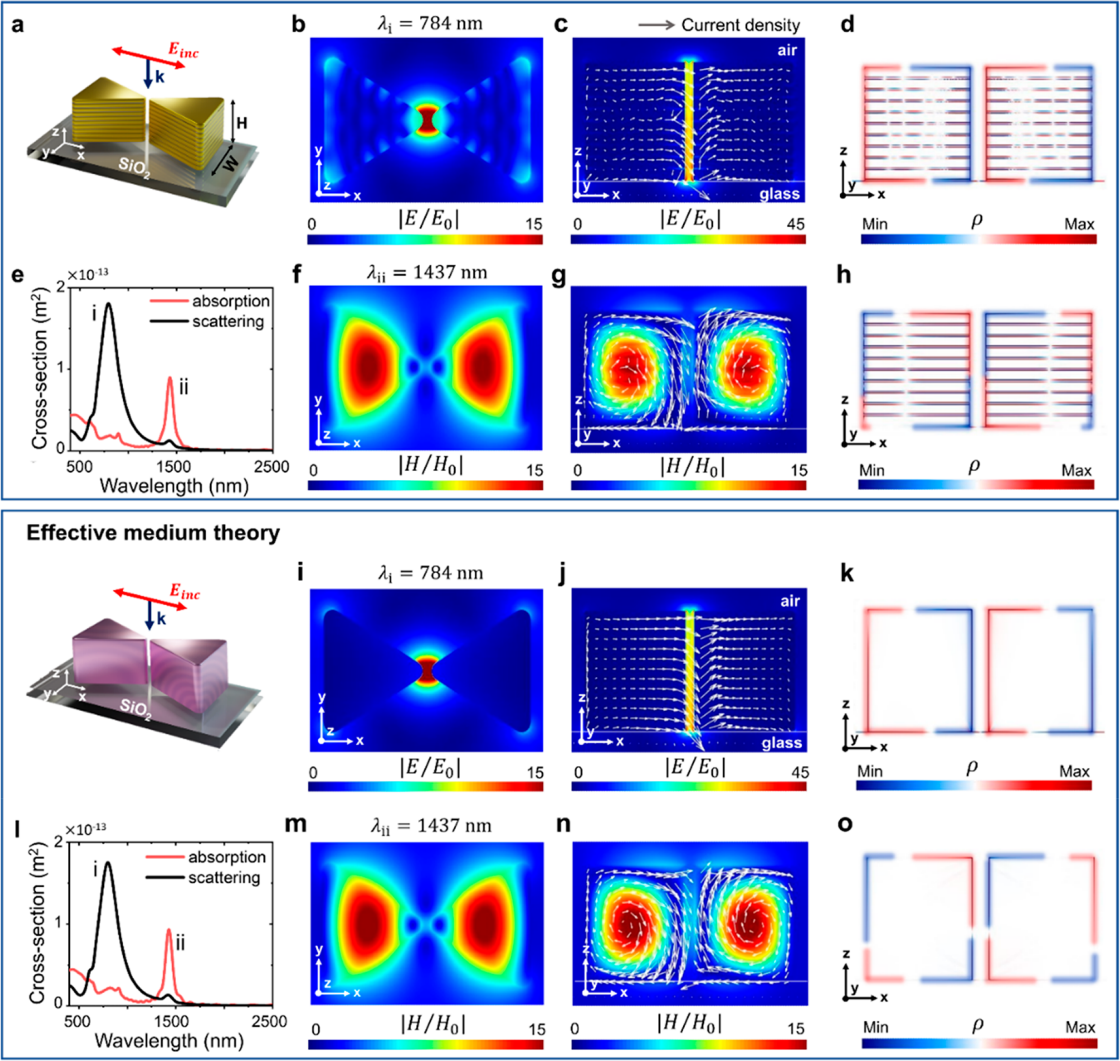
Mode structure supported by the full multilayer gold-TiO_2_ bowtie meta-antenna (*f*_Au_ = 0.83) (a–h) and equivalent meta-antenna with effective permittivity under *x* polarized illumination (i–o). (a) Schematic of the multilayer gold–TiO_2_ bowtie meta-antenna. (b–d) Electric mode (i) at 784 nm: electric field enhancement in the *x*–*y* plane (b), current densities superimposed on the electric field enhancement in the *x*–*z* plane (c), and surface charge distribution (d). (e) Calculated optical cross sections of multilayer meta-antenna. (f–h) Magnetic mode (ii) at 1437 nm: magnetic field enhancement in the *x*–*y* plane (f), current densities superimposed on the magnetic field enhancement in the *x*–*z* plane (g), and surface charge distributions (h). (i–k) Electric mode (i) at 784 nm of effective medium theory (EMT) meta-antenna: electric field enhancement in *x*–*y* plane (i), current densities superimposed on the electric field enhancement in *x*–*z* plane (j), and surface charge distribution (k). (l) Calculated optical cross sections of EMT meta-antenna. (m–o) Magnetic mode (ii) at 1437 nm: magnetic field enhancement in *x*–*y* plane (m), current densities superimposed on the magnetic field enhancement in *x*–*z* plane (n), and surface charge distributions (o). The lines corresponding to the edges of the meta-antenna are graphically enhanced for the surface charge distributions (fourth column) to underline the nature of the modes.

Whereas electric field enhancement has been explored in stratified nanostructures^38^ for surface-enhanced Raman scattering applications, here we report for the first time on the coupling between the magnetic resonances within a dimer hyperbolic meta-antenna, revealing novel coupling mechanisms between hyperbolic systems that have not been reported before, with a detailed study of the magnetic coupling nature, as a function of the structural parameters of the hyperbolic meta-antenna. By modifying the metamaterial fill factor, gap size, and incident polarization, we demonstrate full control over the coupling strength between the magnetic modes and therefore the optical response of the meta-antenna. The presence of coupled magnetic modes in such systems opens the route toward a range of applications, including magnetic nanolight sources, chiral quantum optics, magnetic forces engineering, active control of metamaterial nanostructures, and quantum interfaces for nonreciprocal processing of light.^39−41^ In parallel, the electric dipolar coupling in the meta-antenna results in a significant field enhancement spatially distributed along the height of the nanogap (Figure 1c). We show that the metamaterial fill factor offers a fine control over the spatial distribution of the electrical field enhancement, making meta-antennas suitable candidates for a variety of applications in optical sensors, single photon sources, and subwavelength meta-cavity lasers.

In addition to an in-depth study of the optical properties of meta-antennas, experimental considerations are taken into account in order to map a route toward the fabrication and characterization of this novel coupled meta-antenna geometry.

A typical bowtie geometry^42−45^ is considered to study mode coupling in hyperbolic meta-antennas. The bowtie consists of two equilateral triangular elements positioned tip-to-tip (Figure 1a) with a 10 nm radius of curvature rounding for the corners and edges. The meta-antenna is made from a hyperbolic medium consisting of a gold and TiO_2_ multilayer system on a glass substrate. The width (*w*) of each triangle, along with the total height (*H*) of the structure, is kept constant throughout the study, with *w* = 133 nm and *H* = 120 nm.

In a first instance, we consider a meta-antenna with a gap of 10 nm and a multilayer metamaterial with metal fill factor of 0.83, corresponding to a gold and TiO_2_ thickness of *t*_Au_ = 10 nm and *t*_TiO_2__ = 2 nm, respectively. The resulting uniaxial effective permittivity tensor has a type II hyperbolic dispersion (ε_∥_ < 0, ε_⊥_ > 0) for wavelengths longer than 780 nm leading to the well-known onefold hyperboloid iso-frequency surface (Figure S1, Supporting Information).^7,8,21^

The resulting coupled hyperbolic nanostructure supports two different resonances, clearly identified by the scattering and absorption spectra of the hyperbolic meta-antenna (Figure 1e): a mainly scattering mode (i) at a wavelength of 784 nm and a strongly absorbing mode (ii) at a longer wavelength of 1437 nm. The spatial field distributions, charge densities (ρ), and current densities (*J*) for each mode allow us to determine the nature of those optical resonances (Figure 1b–d,f–h). The current density directed along the bowtie meta-antenna long axis (*x*-axis) and surface charge distribution (Figure 1c,d) for mode (i) identify this resonance as a bonding dimer mode arising from the coupling between the electric dipolar resonances of each triangular elements of the meta-antenna,^46−51^ resulting in strong near-field enhancement in the gap region. It is interesting to compare this mode to the equivalent pure gold bowtie nanoantenna, which supports a similar scattering mode (Figure S2, Supporting Information). In the case of a purely metallic nanoantenna, interaction with the substrate results in a nonzero vertical component of the current displacement, leading to a well-known inhomogeneous field enhancement in the gap^46^ with the highest intensity confined near the substrate (see Figure S2d, Supporting Information). Interestingly, due to the high perpendicular component of the effective refractive index at this resonance (Figure S8, Supporting Information), the electric dipolar mode of the meta-antenna is unaffected by the presence of the glass substrate, resulting in a homogeneous field enhancement across the gap height (Figure 1c). Such homogeneous distribution of the electric field enhancement inside the gap is highly desirable in many light–matter applications,^46^ such as sensing, single photon sources, and subwavelength meta-cavity lasers, where the analytes or quantum emitter need to be positioned in the field maximum.

Mode (ii), on the other hand, is linked to closed loops in the current densities and a confined magnetic field inside each triangular element of the meta-antenna (Figure 1f,g), clearly corresponding to the excitation of magnetic dipoles in elements of the meta-antenna. Although this magnetic mode has been previously observed in cylindrical metaparticles,^16,52^ it is important to consider the shape effect on the magnetic resonance of a single element of the bowtie. The triangular geometry considered here clearly supports the magnetic resonance at a wavelength of 1406 nm, with the spectral position of the mode independent of the incident light polarization (Figure S3a, Supporting Information). The corresponding induced magnetic dipole moments lie in the *x*–*y* plane and are oriented perpendicular to the incident polarization (see Figure S3c,d, Supporting Information). However, in the case of the bowtie meta-antenna, the close proximity of the two elements leads to an overlap of the current density loops, resulting in a coupling of the magnetic dipolar resonances. This coupling between the magnetic dipole moments results in a redshift of the resonance wavelength when compared to the isolated triangle (see Figure S3a, Supporting Information). Moreover, the increased circular displacement currents in the gap result in an additional magnetic field in the center of the nanogap, with the corresponding electric field for this mode (Figure S4, Supporting Information) concentrated at the top and bottom of the nanogap. Calculations show that the magnetic mode is not supported for structures with less than four metal–dielectric bilayers, and increasing the number of bilayers results in a more defined mode with a larger quality factor (Figure S5, Supporting Information).

In parallel to the exact multilayer geometry (Figure 1e), full-wave numerical calculations of the hyperbolic bowtie meta-antenna with an equivalent homogeneous anisotropic permittivity tensor based on effective medium theory (EMT)^33,36^ were performed. The scattering/absorption spectra, charge distributions, and current densities as well as the electric and magnetic field distributions of the effective medium meta-antenna show a perfect agreement with the full multilayer meta-antenna calculations (Figure 1i–o, Figure S5, Supporting Information), not only confirming the intrinsic hyperbolic behavior of the meta-antenna but also further validating the nature of the modes.

The fill factor (*f*_Au_) in the multilayer system allows a fine-tuning of the metamaterial optical properties and therefore directly impacts the spectral position, intensity, and local-field enhancement of the modes as well as their coupling in the meta-antenna. Plotting the function *Re*[ε_⊥_]·*Re*[ε_∥_] allows identification of the hyperbolic region (*Re*[ε_⊥_]·*Re*[ε_∥_] < 0) along with the dielectric (*Re*[ε_⊥_] > 0 and *Re*[ε_∥_] > 0) and metallic (*Re*[ε_⊥_] < 0 and *Re*[ε_∥_] < 0) regions (Figure 2a) for different fill factors. To further explore the coupling of the electric and magnetic resonances, the optical properties of the meta-antenna were calculated (Figure 2b–d) with effective medium theory for a fill factor in the range 0 ≤ *f*_Au_ ≤ 1, with *f*_Au_ = 0 corresponding to a purely dielectric regime (100% TiO_2_ bowtie antenna) and *f*_Au_ = 1 referring to a metallic antenna (100% gold bowtie). Overlaying the spectral position of the exact multilayer meta-antenna resonances (individual points in Figure 2b–d) shows a perfect agreement between the effective medium theory and the full multilayer geometry across the whole range of fill factors.

**Figure 2:**
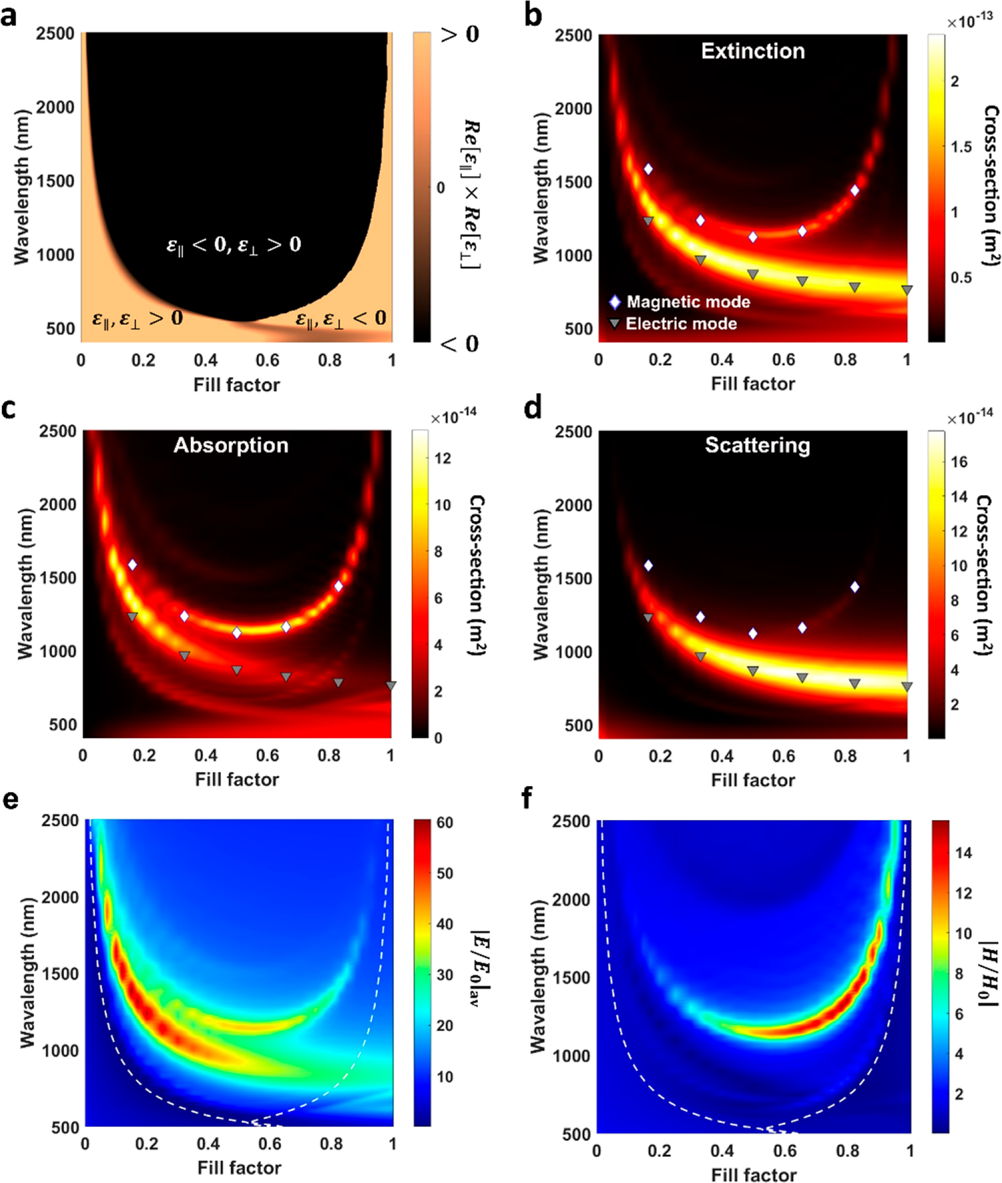
(a) Hyperbolic phase diagram predicted by EMT as a function of fill factor. The orange regions represent an elliptical dispersion (metallic or dielectric). The black region indicates the hyperbolic dispersion, where the function *Re*[ε_⊥_]·*Re*[ε_∥_] is negative. The color maps show the extinction (b), absorption (c), and scattering (d) cross sections as a function of gold fill factor for the hyperbolic effective medium meta-antennas. Individual points show the positions of the corresponding resonances for the full multilayer bowtie meta-antennas. (e) Averaged electric field enhancement in the meta-antenna nanogap as a function of fill factor and wavelength. (f) Magnetic field enhancement in the middle of one element of the meta-antenna as a function of fill factor and wavelength.

The electric dipolar mode is shown to exist in both the metallic and hyperbolic regions, only bound by the hyperbolic region for the low fill factor values (Figure 2e). As the fill factor increases, the electric dipolar mode blue-shifts by more than 1500 nm, from >2000 to 764 nm, converging onto the pure gold resonance for *f*_Au_ = 1. It is important to note that the proportion of the mode inside the metamaterial is higher for lower fill factors (Figure S6, Supporting Information), slowly decreasing as the parallel component of the effective permittivity becomes more negative at higher fill factors. This results in both a higher electric field in the gap, and a more absorbing behavior at lower fill factors. The higher field in the gap arises from the stronger field inside the metamaterial and the continuity of the normal component of the electric displacement field at the boundary. In parallel, as lower fill factors correspond to a larger proportion of the mode volume inside the metamaterial, with a nonzero imaginary part of the effective permittivity, the electric dipolar mode behavior changes from highly scattering at high fill factors to more absorbing for the lower fill factors. Additionally, the electric field spatial distribution along the height of the nanogap can be engineered by tuning the fill factor (Figure S7, Supporting Information). This fine control over the electrical field enhancement highlights the versatility of meta-antennas for electric gap mode engineering.

The magnetic mode, on the other hand, exists solely in the hyperbolic region and is bound by the hyperbolic dispersion (Figure 2b). As the fill factor increases, the mode initially blue-shifts and then reaches the minimum wavelength of 1116 nm for *f*_Au_ = 0.5 and turns to a longer wavelength for a higher fill factor. Consequently, the magnetic dipolar mode spectral position is more sensitive to variations in fill factor for its extreme values (*f*_Au_ > 0.85 or *f*_Au_ < 0.15), whereas for 0.3 < *f*_Au_ < 0.7, changes in the fill factor result in near negligible variations of the magnetic mode spectral position. Additionally, the magnetic mode intensity is not constant across the range of fill factors, with larger fill factors corresponding to higher magnetic field amplitudes inside the meta-antenna elements (Figure 2f). This increase of the magnetic mode intensity with fill factor is directly connected to higher values of the perpendicular component (*z* direction) of the metamaterial effective index (Figure S8, Supporting Information).^36^ The evolution of the electric and magnetic dipolar modes as a function of the fill factor presented here can inform future meta-antenna design, fabrication, and technological development.

Although the optical modes for the isolated triangular metaparticle also depend on the fill factor (see Figure S3b, Supporting Information), the coupling of the magnetic modes in the bowtie meta-antenna modifies their spectral position, resulting in a clear redshift (Figure 3a), therefore providing a powerful handle on their magnetic resonances. Interestingly, this spectral shift decreases with increasing fill factor and slowly disappears for *f*_Au_ > 0.85, indicating a change in the coupling strength as a function of the material properties.

**Figure 3:**
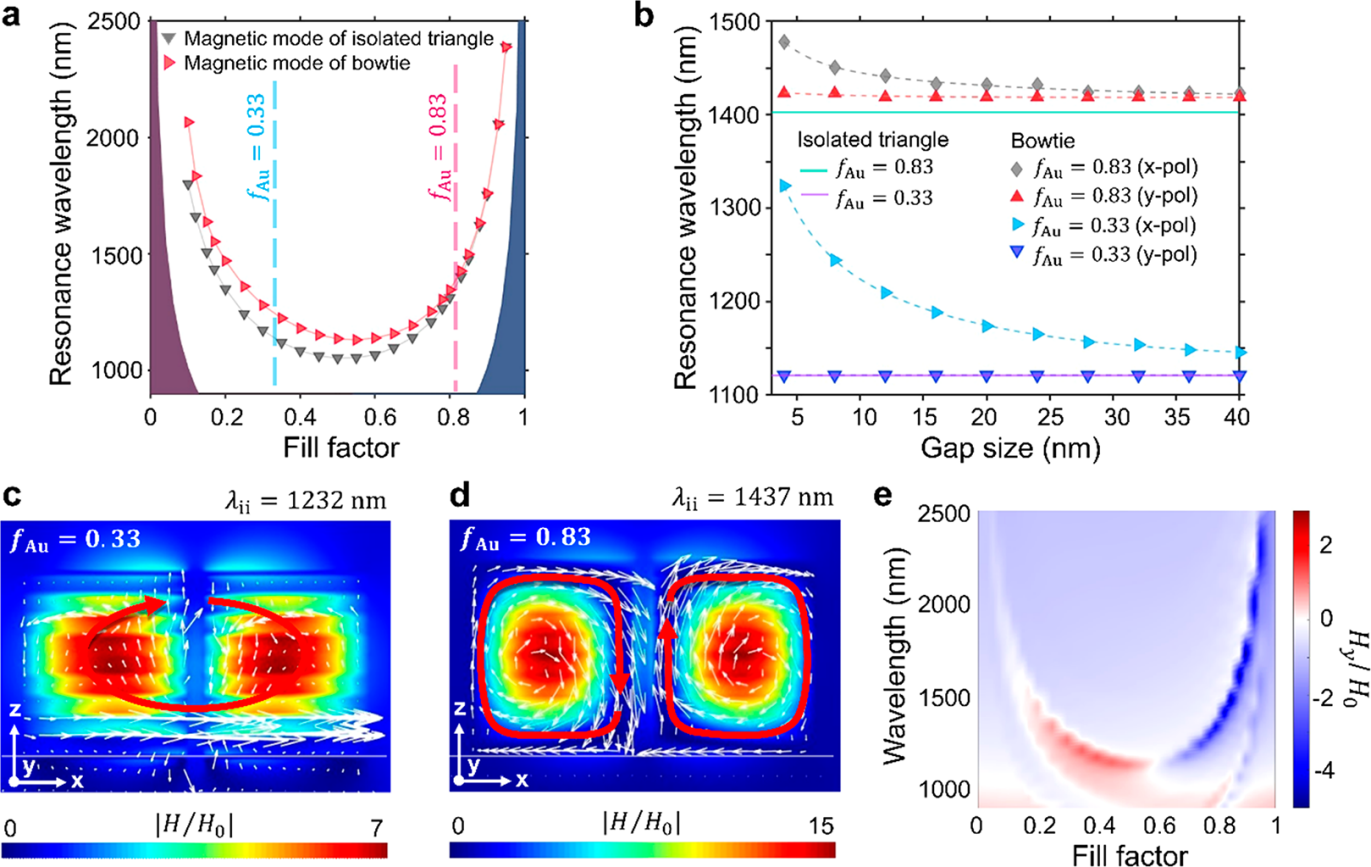
(a) Magnetic mode spectral position as a function of fill factor for an individual metaparticle and bowtie meta-antenna. Dark parts underline the end of the hyperbolic region. (b) Magnetic mode spectral position as a function of meta-antenna gap size and incident polarization for *f*_Au_ = 0.83 and *f*_Au_ = 0.33. (c,d) Current densities superimposed on the magnetic field enhancement maps in the *x*–*z* plane for meta-antennas with *f*_Au_ = 0.33 and *f*_Au_ = 0.83. (e) Real part of *y* component of the magnetic field, *H*_*y*_, in the meta-antenna nanogap as a function of fill factor and wavelength for a gap size of 10 nm.

To study this coupling in more depth, the gap separating the individual elements in the meta-antenna was varied from 4 to 40 nm. The spectral position of the magnetic mode for two representative fill factors, 0.83 and 0.33, was plotted as a function of gap size and incident polarization (Figure 3b). As opposed to the isolated triangle structure (see Figure S3, Supporting Information), the magnetic mode behavior for the coupled meta-antenna is clearly polarization dependent. Under *y* polarization, the spectral position is completely independent of the gap size and remains at a fixed value. This is expected as this polarization results in individual magnetic dipoles oriented in the *x*-direction, arising from current loops positioned in the *y*–*z* plane, which, along with the triangular shape of the individual meta-antenna elements, results in the magnetic dipole moment positioned at the back of the triangular element away from the nanogap, leading to an absence of coupling between the two individual bowtie elements (see Figures S3 and S9, Supporting Information).

On the other hand, for *x* polarization, the spectral position of the magnetic mode changes exponentially with decreasing gap size, red-shifting by 55 and 180 nm for fill factors of 0.83 and 0.33, respectively. Additionally, for the larger gap sizes, the spectral position of the magnetic mode for both polarizations almost overlap and converge toward the isolated triangle of the same fill factor. This clearly illustrates the coupling of the magnetic modes in the meta-antenna for a polarization along the axis of the dimer (*x* polarization).

The nature of the interaction, however, varies as a function of the fill factor. In this case, the current density loops are in the *x*–*z* plane, resulting in magnetic dipole moments along the *y*-direction, perpendicular to the axis of the meta-antenna. For *f*_Au_ = 0.83, the current density loops from each half of the meta-antenna are separated, resulting in counter-propagating current densities inside the nanogap (Figure 3d). This current configuration between the two individual elements results in an additional localized magnetic field maximum inside the nanogap. Due to the resulting current orientation, the induced magnetic field is oriented in the opposite direction compared to the field inside the metamaterial. Additionally, the high confinement of the magnetic modes for this fill factor means that the coupling is observed only for gap sizes below 25 nm. For larger gaps, the confinement of the current density loops inside each individual element of the meta-antenna prevents any interaction between them.

For *f*_Au_ = 0.33, on the other hand, the magnetic modes are less confined inside the triangular elements, resulting in a larger interaction of the current densities inside the meta-antenna, ultimately forming a single loop across the whole nanostructure (Figure 3c). This gives rise to a coupled mode encompassing both parts of the meta-antenna, forming a large current density loop and resulting in the larger redshift (1145 to 1325 nm) observed for this fill factor with decreasing gap size. Consequently, the magnetic field inside the meta-antenna nanogap is oriented in the same direction as that of the original individual magnetic dipoles.

This change in the coupling nature, and therefore reversal of the magnetic field direction inside the gap, is clearly visible by considering the sign of the *y* component of the magnetic field, *H*_*y*_, as a function of the fill factor. For example, a meta-antenna with a 10 nm gap shows a clear sign reversal in *H*_*y*_ for a fill factor of *f*_Au_ = 0.6 (Figure 3e). Accordingly, *f*_Au_ < 0.6 corresponds to a magnetic coupling resulting in a collective current density loop and a magnetic field in the gap oriented in the same direction as the original individual magnetic dipoles. Correspondingly, this type of coupling results in larger spectral shifts in magnetic resonance, when compared to the isolated triangular metaparticle. Conversely, for *f*_Au_ > 0.6, the higher confinement of the magnetic modes leads to a coupling based on counter-propagating current densities and a reversal magnetic field direction in the nanogap corresponding to a lower spectral shift, which eventually disappears completely as the modes are fully confined inside the material (Figure 3a). The fill factor therefore offers a powerful means of controlling the nature of magnetic coupling inside the meta-antenna.

An alternative way to probe the coupling of the magnetic modes is to modify the meta-antenna effective index by keeping the metal fill factor constant and varying the refractive index (RI) of the dielectric used in the multilayer system (Figure 4). The RI of the dielectric layers was therefore changed between *n* = 1 and *n* = 4, and the resulting effective permittivity was calculated accordingly. It is important to note that increasing the RI of the dielectric results in a modification of the metamaterial hyperbolic dispersion, therefore shifting the hyperbolic region to the near-infrared region, as illustrated in Figure 4a,b. The magnetic mode spectral position shifts accordingly and remains in the hyperbolic region. As expected, the position of the mode for the isolated triangle varies with the RI, as the effective permittivity of the multilayer changes. In the bowtie meta-antenna, for large refractive indices of the dielectric, the magnetic mode is confined within the meta-antenna individual element with minimal field outside of the metamaterial, as illustrated by the current density loops (Figure S10, Supporting Information). This results in greatly reduced interaction between the elements of the bowtie and an overlap of the resonances spectral position with the isolated triangle (Figure 4a,b). For high RI systems, the coupling is limited and mainly associated with the counter-propagating current densities in the nanogap linked to a reversal of the magnetic field direction (Figure 4, insets). However, for lower refractive indices, the response of the meta-antenna diverges from that of the isolated triangles: as the RI decreases, the magnetic mode is less confined inside the metamaterial, and a larger proportion of the magnetic field extends into the nanogap, leading to an increased coupling and a corresponding redshift of the optical response.

**Figure 4:**
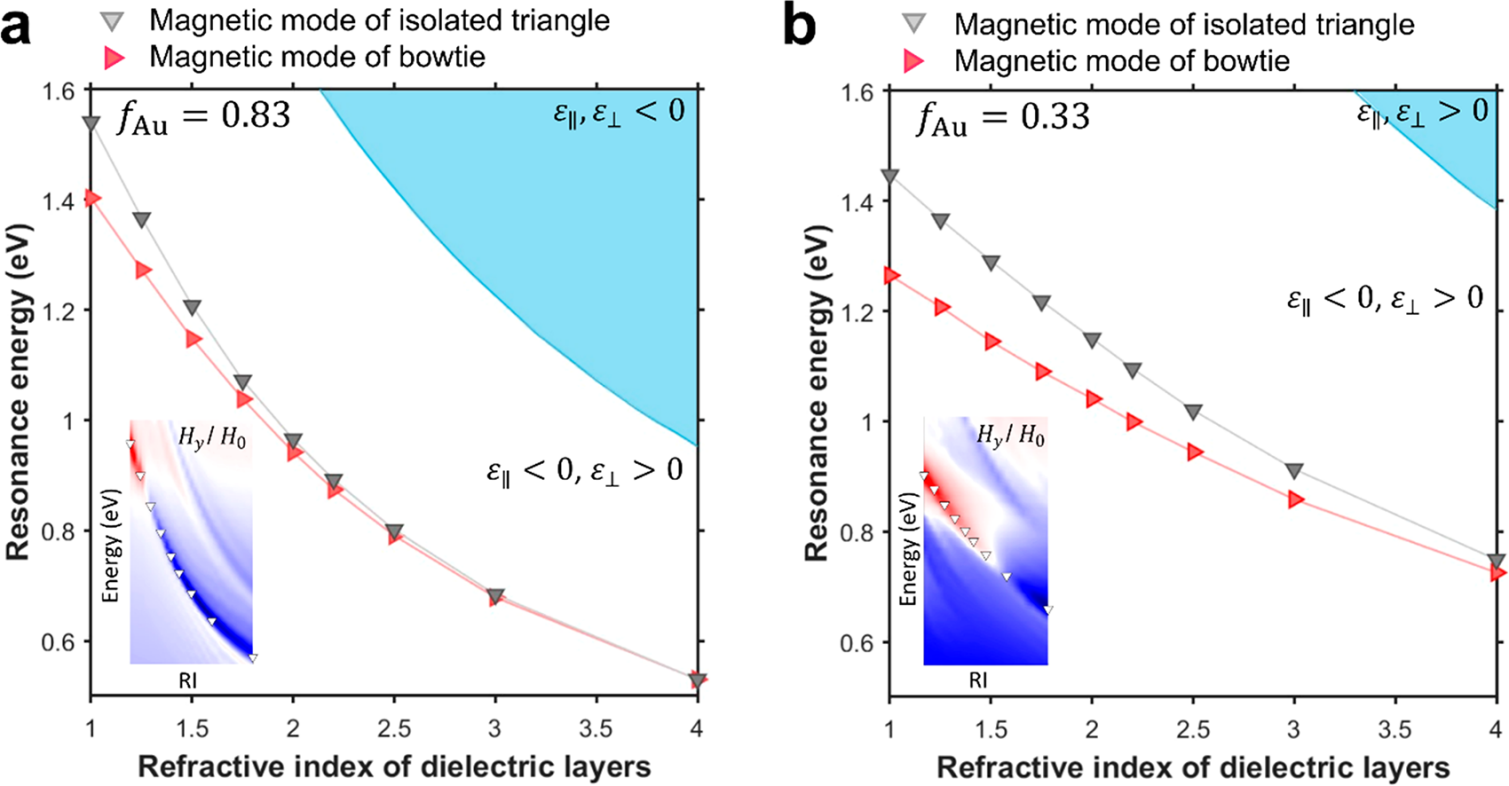
Magnetic mode spectral position as a function of the dielectric refractive index in the metal-dielectric multilayer system for the bowtie meta-antenna and the isolated triangular metaparticle with *f*_Au_ = 0.83 (a) and *f*_Au_ = 0.33 (b). Light blue (cyan) areas outline the nonhyperbolic region. Insets show the real part of *y* component of the magnetic field, *H*_*y*_, in the meta-antennas nanogap as a function of the dielectric refractive index in the multilayer system.

The larger spectral shift observed for the lower values of the dielectric refractive index (Figure 4a,b) confirms the magnetic coupling behavior in meta-antennas discussed above. The reduced magnetic mode confinement for low dielectric RI systems facilitates the collective coupling, resulting in one effective loop, over a larger range of fill factors (Figure 4b, inset). Correspondingly, the shift between the two magnetic coupling regimes occurs at lower values of the dielectric RI for higher fill factors (Figure 4, insets).

Therefore, depending on the desired parameters (spectral region, coupling strength, magnetic mode intensity in the gap and inside the metaparticles, and electric field confinement in the gap), the properties of the meta-antenna can be controlled in multiple ways during the nanofabrication process. The effective coupling of the magnetic modes occurs for experimentally relevant gap sizes of up to 20 nm. The spectral positions of the coupled electric and magnetic modes were shown to be stable across a wide range of fill factors, making the meta-antenna design robust to small experimental variations of the dielectric constants of the materials used during the nanofabrication process. Additionally, the perfect agreement shown between the full multilayer geometry and the homogeneous effective medium structure promises some freedom in the meta-antenna multilayer design, where the layer thicknesses can be modified to suit nanofabrication capabilities as long as they result in similar effective permittivity.

In conclusion, we have introduced a new class of hyperbolic systems based on coupled metaparticles to create a meta-antenna supporting the excitation of electric and magnetic dipolar modes, the spectral position of which can be tuned from the visible to the infrared regions through material effective permittivity engineering. We have shown that the coupling of hyperbolic nanostructures creates a rich mode structure that greatly enlarges the design opportunities for engineering light–matter interactions on the nanoscale and clearly evidenced the coupling of the electric and magnetic resonances in such meta-antennas and illustrated its dependence on the meta-antenna effective permittivity.

In parallel to an electric field enhancement distributed along the height of the nanogap, promising for applications in optical sensors, single photon sources, and subwavelength meta-cavity lasers, the meta-antennas were shown to support two distinct magnetic coupling regimes, depending on the interplay of the magnetic dipole moments, controllable by the meta-antenna effective permittivity, gap size, and incident polarization. This newly reported dual nature of the magnetic coupling and its dependence on the structural parameters of the hyperbolic meta-antenna opens the route toward a range of applications, including magnetic nanolight sources, chiral quantum optics, magnetic forces engineering, active control of metamaterial nanostructures, and quantum interfaces for nonreciprocal processing of light.

Although challenging to fabricate, such structures can be realized using a combination of multilayer deposition and directional etching, using either reactive ion etching through a bimetallic mask created via electron beam or nanoimprint lithography, or alternatively by directly milling the metal–dielectric multilayer using a focused ion beam.

## Methods

Numerical investigations based on the commercial 3D finite-difference time-domain (FDTD) method from Lumerical-FDTD (Ansys) have been performed using a normal incidence total-field scattered-field (TF/SF) source and perfectly matched layer (PML) boundary conditions over the wavelength range from 400 to 2500 nm, with a 0.5 nm non-conformal mesh surrounding the meta-antenna region. The calculations were terminated when the fields had decayed below 10^–5^ of their original value. The meta-antenna consists of a bowtie geometry with two equilateral triangular elements positioned tip-to-tip (Figure 1a) with a 10 nm radius of curvature rounding for the corners and edges, made from a hyperbolic medium consisting of a gold and TiO_2_ multilayer system on a glass substrate. The width (*w*) of each triangle and the total height (*H*) of the structure are kept constant throughout the study, with *w* = 133 nm and *H* = 120 nm. The surrounding medium, glass substrate, and TiO_2_ are considered dispersionless with refractive indices of 1, 1.47, and 2.2 respectively, while the material parameters for gold are taken from Johnson and Christy.^53^ The metal fill factor is defined as *f*_Au_ = *t*_Au_/(*t*_Au_ + *t*_TiO_2__) where *t*_Au_ and *t*_TiO_2__ are the thicknesses of individual gold and TiO_2_ layers, and the corresponding effective permittivity components for the gold-TiO_2_ multilayer metamaterial were determined via effective medium theory (EMT). The effective permittivity tensor was calculated using ε_∥_(ε_*xx*_ = ε_*yy*_) = *f*_Au_ε_Au_ + (1 – *f*_Au_)ε_d_ and ε_⊥_(ε_*zz*_) = [ε_Au_ε_d_]/[(1 – *f*_Au_)ε_Au_ – *f*_Au_ε_d_], where ε_Au_ and ε_d_ describe the relative permittivity of the metal and dielectric layers, respectively (see Supplementary Note S1 for more details).

The extinction spectra were calculated as the sum of the scattering spectrum and the absorption spectrum. Averaged electric field enhancements were calculated by integrating the field in the gap along the meta-antenna height and averaging correspondingly. The surface charge distributions and current densities used to identify the modes and their coupling were calculated according to previously described formalisms.^54^

While we discuss the experimental feasibility of the introduced geometry in this work, the effects of roughness, dielectric constant variability, defects due to nanofabrication, crystallization, and nonlocality were not considered.^33,55,56^

## Supporting Information Available

Calculations of the effective permittivity using the effective medium theory, optical characterization of the Au bowtie nanoantenna, optical characterization of a single metaparticle (triangular nanoprism), electric near-field enhancement of the hyperbolic multilayer in *xz* and *xy* cuts, dependence of the optical properties of the meta-antenna on height, electric field distribution inside and outside the meta-antenna, the line-scans of the normalized electric field across the height of the nanogap as a function of the wavelength, refractive index as a function of gold fill factor for the hyperbolic effective medium, magnetic field enhancement in *x*–*z* plane for the bowtie meta-antenna, the current densities superimposed on magnetic field-amplitude distributions of the magnetic modes for (a) bowtie meta-antennas and (b) individual metaparticles (PDF)
