# Supplementary material for: Magnetic Mode Coupling in Hyperbolic Bowtie Meta-Antennas
Source: J Phys Chem Lett. 2023 Aug 25;14(35):7824–32. doi: 10.1021/acs.jpclett.3c01620 (PMC10494229; doi:10.1021/acs.jpclett.3c01620)
Supplement: Supplementary file 1 — jz3c01620_si_001.pdf [file jz3c01620_si_001.pdf]

Magnetic Mode Coupling in Hyperbolic Bowtie Meta-Antennas

Sema Ebrahimi,[1,2,3,,†] Alina Muravitskaya[2,3,†] Ali M. Adawi,[2,3] Anne-Laure Baudrion,[1] Pierre-Michel Adam,[1,*] and Jean-Sebastien G. Bouillard[2,3,*]*

[1] Light, nanomaterials and nanotechnologies Laboratory, CNRS EMR 7004, University of Technology of Troyes, F-10004 Troyes Cedex, France
[2] Department of Physics and Mathematics, University of Hull, Cottingham Road, Hull, HU6 7RX, UK
[3] G.W .Gray Centre for Advanced Materials, University of Hull, Cottingham Road, Hull, HU6 7RX, UK
† These authors contributed equally to this work.
* Sema Ebrahimi, E-mail: semaebrahimi@gmail.com,
Pierre-Michel Adam, E-mail: pierre_michel.adam@utt.fr,
Jean-Sebastien G. Bouillard, E-mail: j.bouillard@hull.ac.uk

Supplementary Note S1: Calculation of the Effective Medium Theory (EMT) for a multilayer system of Au/ TiO_2 bowtie meta-antennas.

Using effective medium approach, we calculated the optical response of the composite medium made of alternating layers of metal (Au) and dielectric (TiO_2) (Figure S1a). Bruggeman's effective medium theory considers mixture of two materials and can be applied for the composites with arbitrary volume fractions. According to the literature, the hyperbolic metamaterials serve as a uniaxial medium with permittivity given by a tensor $\varepsilon = [\varepsilon_{xx}, \varepsilon_{yy}, \varepsilon_{zz}]$, where the in-plane components are defined as $\varepsilon_{xx} = \varepsilon_{yy} = \varepsilon_{\parallel}$, and the out-of-the-plane component describes by $\varepsilon_{zz} = \varepsilon_{\perp}$. Then, we were looking for the effective permittivity tensor of the Au/TiO_2 multilayer system depending on Au fill factor:

$$f_{Au} = \frac{d_m}{d_m + d_d} \quad \text{(S1)}$$

where d_m and d_d are the total thickness of metal and dielectric layers, respectively. Given generalized Maxwell's Equations and using electromagnetic field boundary conditions, the parallel and perpendicular components of the effective permittivity in a multilayer system can be written as follows:

$$\varepsilon_{\parallel}(\varepsilon_{xx} = \varepsilon_{yy}) = f_{Au}\varepsilon_{Au} + (1 - f_{Au})\varepsilon_d \quad \text{(S2)}$$

$$\varepsilon_{\perp}(\varepsilon_{zz}) = \frac{\varepsilon_{Au}\varepsilon_d}{(1-f_{Au})\varepsilon_{Au} - f_{Au}\varepsilon_d} \quad \text{(S3)}$$

where ε_{Au} and ε_d describe the relative permittivity of the metal and dielectric layers, respectively. As represented in Figure S1(b), the effective permittivity of the Au/TiO_2 multilayer structure of $f_{Au} = 0.83$ is different from that of pure gold, revealing the hyperbolic behavior in the region >780 nm.

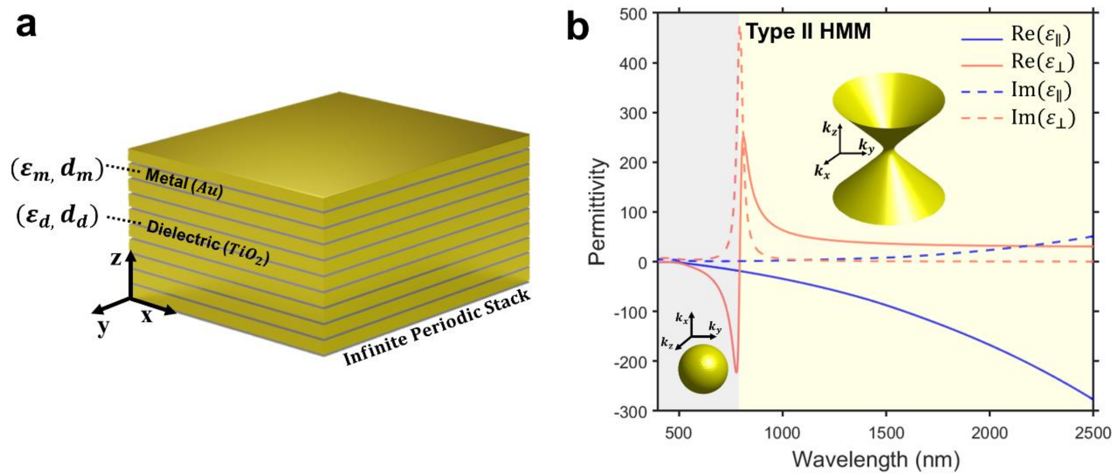

Figure S1. (a) Schematic illustration of hyperbolic multilayer system made of ten alternating layers of Au and TiO_2. (b) The real and imaginary parts of the effective permittivity for the Au/TiO_2 multilayer nanostructure ($f_{\mathrm{Au}} = 0.83$) determined by effective media theory (EMT).

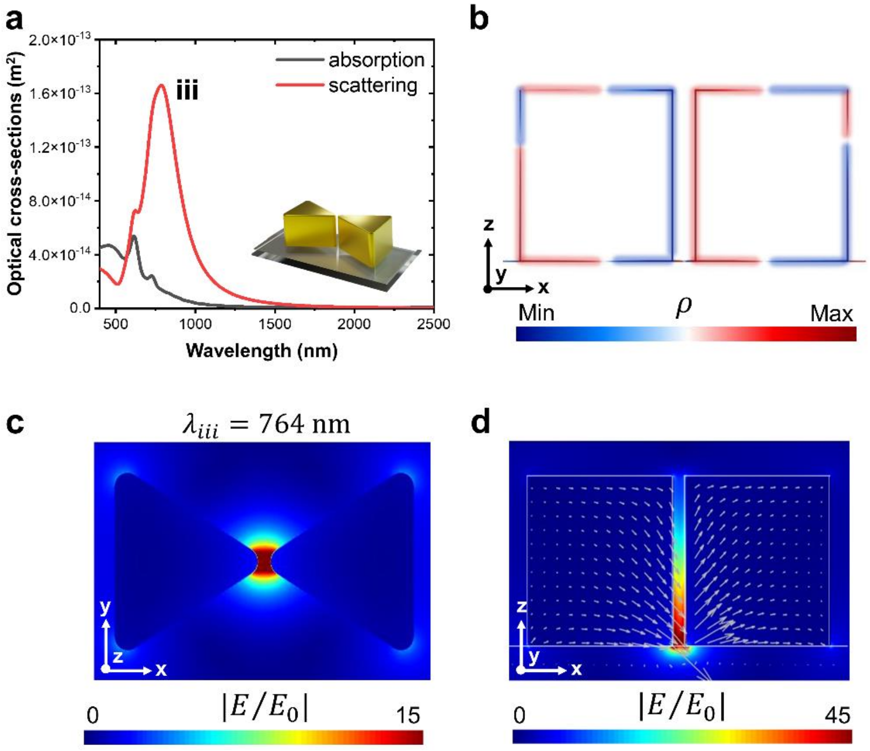

Figure S2. (a) Calculated optical cross sections of Au bowtie nanoantenna with the total thickness of 120 nm under x-polarization illumination. Surface charge distribution in x-z plane (b), field enhancement in x-y plane (c), and current densities superimposed on the electric field enhancement in x-z plane (d) for the mode (iii) at 764 nm. The x-y and x-z planes have been plotted at middle height and middle width for each bowtie.

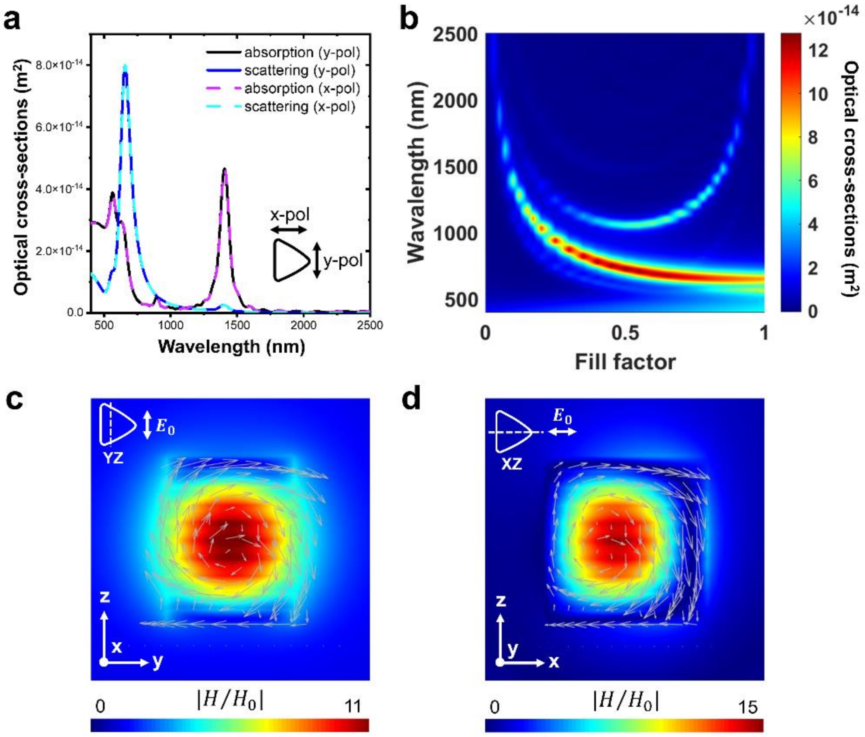

Figure S3. (a) Calculated optical cross sections of metaparticle (triangular nanoprism, f_Au=0.83) under different polarizations. (b) The colormap of extinction cross sections as a function gold fill factor for the hyperbolic metaparticle under x-polarization illumination. (c) Magnetic field enhancement in y-z plane for the magnetic mode under y-polarization. (d) Magnetic field enhancement in x-z plane for the magnetic mode under x-polarization.

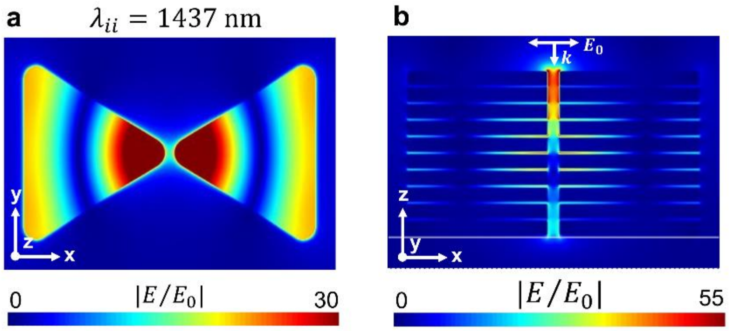

Figure S4. (a, b) Electric near-field enhancement of the hyperbolic multilayer Au/TiO_2 bowtie meta-antenna (f_{Au}=0.83) at magnetic mode wavelength (λ_{ii} = 1437 nm). The x-y and x-z planes have been plotted at middle height and middle width for each bowtie.

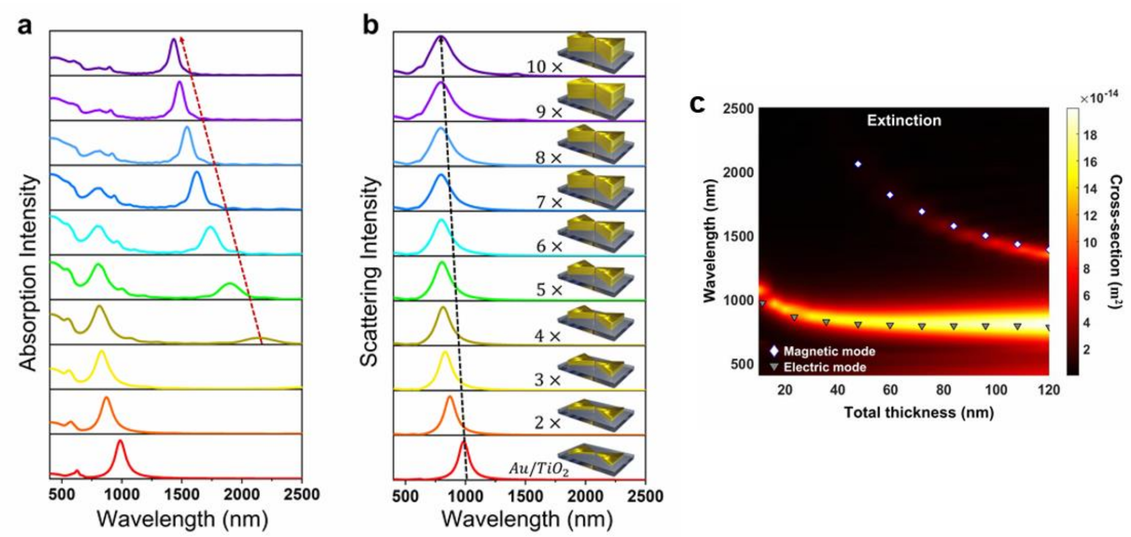

Figure S5. (a) Absorption (a) and scattering (b) intensities and extinction (c) cross-section as a function of structure thickness for a metal fill factor of 0.83. The colormap shows the extinction cross-section as a function of meta-antenna height using EMT. The data points show the positions of the corresponding resonances for the full multilayer bowtie meta-antennas.

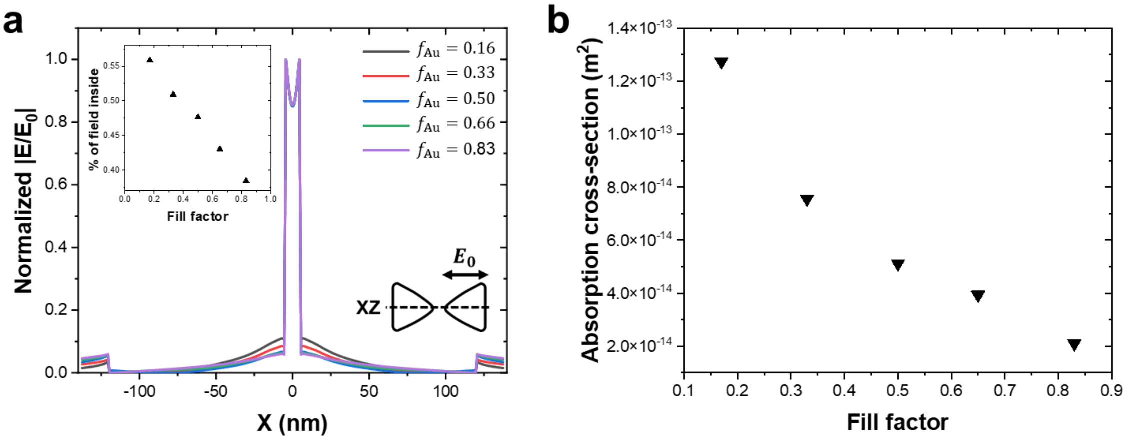

Figure S6. (a) Normalized electric field scan along x axis averaged over the height of meta-antenna for the different fill factors of the metamaterial. Inset: ratio of the electric field inside the elements of the meta-antenna to the total field in scan. (b) Absorption of the electric dipolar mode for the meta-antennas of different fill factors (extracted from Fig. 2c)

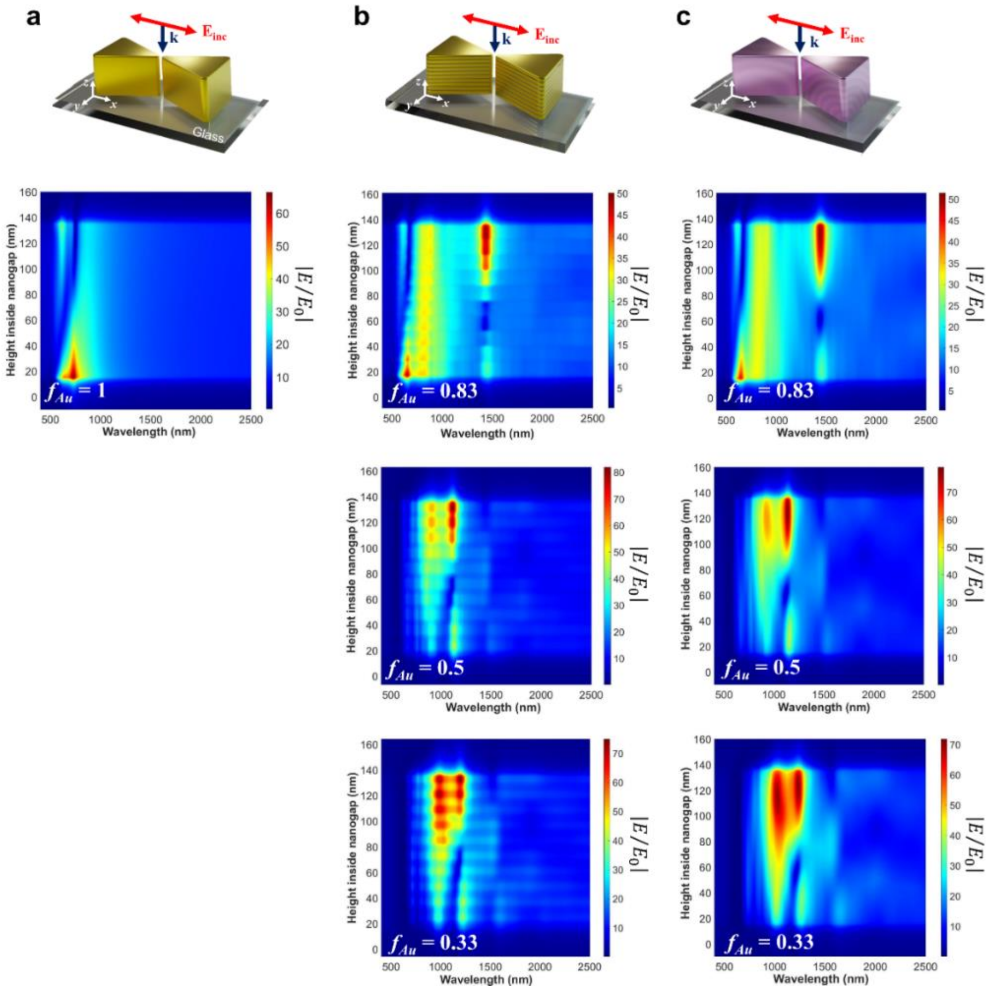

Figure S7. The line-scans of the normalized electric field across the height of the nanogap as a function of the wavelength for (a) Au nanoantennas and (b) hyperbolic bowtie meta-antennas with various Au fill factors and (c) meta-antennas with corresponding homogeneous effective index. The illumination is polarized along x axis.

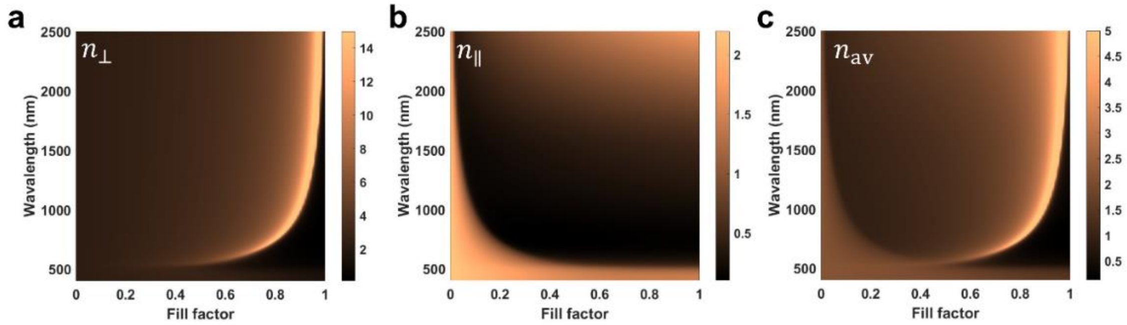

Figure S8. Refractive index as a function of gold fill factor for the hyperbolic effective medium. (a) $n_\perp$ component. (b) $n_\parallel$ component. (c) $n_{av} = (n_\parallel + n_\parallel + n_\perp)/3$ averaged value.

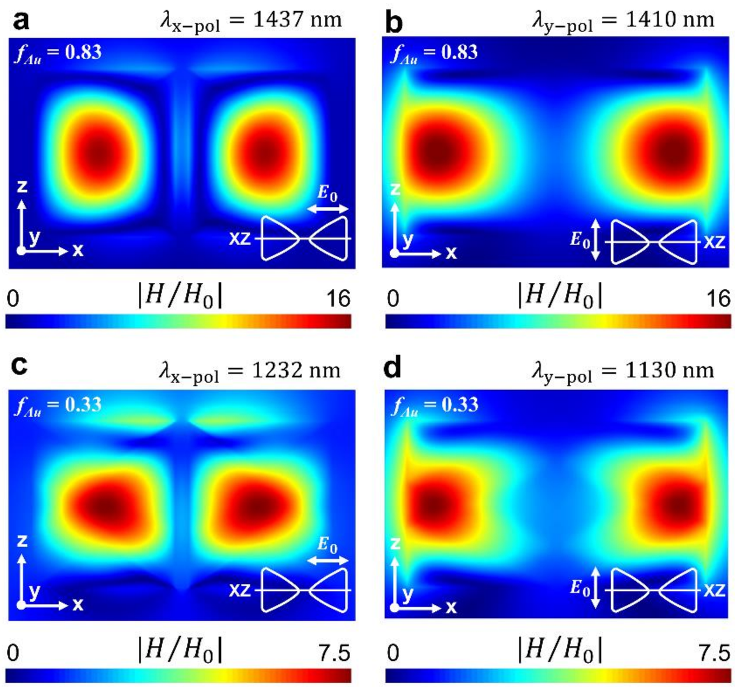

Figure S9. Magnetic field enhancement in x-z plane for the bow-tie meta-antenna with $f_{\text{Au}} = 0.83$ (a,b) and $f_{\text{Au}} = 0.33$ (c,d) under x-polarized (a,c) and y-polarized (b,d) excitation.

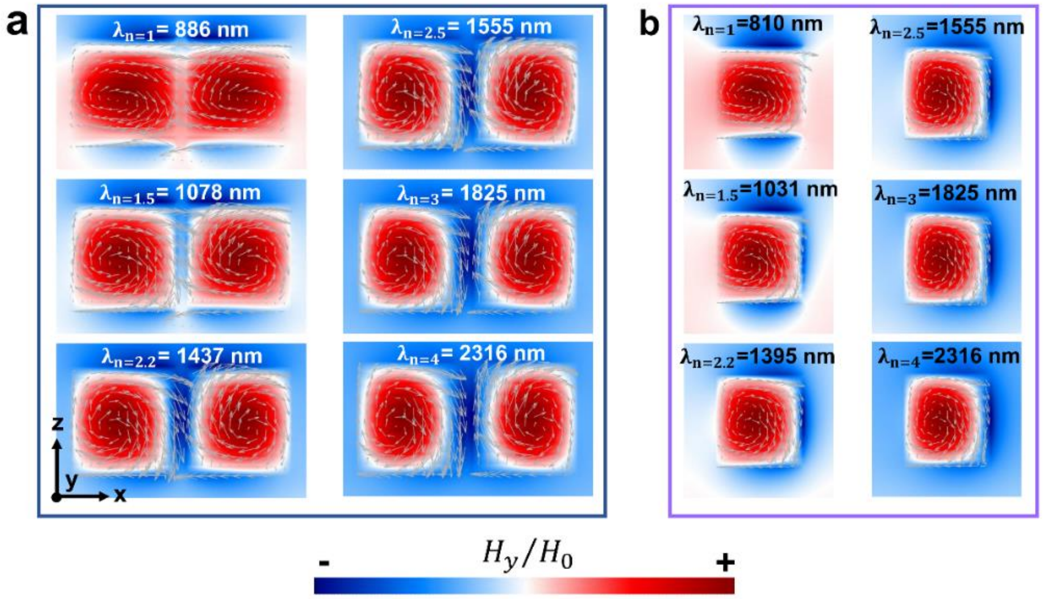

Figure S10. The current densities superimposed on magnetic field-amplitude distributions of the magnetic modes for (a) bowtie meta-antennas and (b) individual metaparticles with the Au fill factor (f_{Au}) of 0.83 at different refractive indices of the dielectric layer from $n = 1$ to $n = 1.5, 2.2, 2.5, 3$ and 4.
